# Tobacco as a Remedy

**Published:** 1885-01-20

**Authors:** 


### TOBACCO AS A REMEDY.

[The following paper is from a non-professional source, being an extract from an article by United States Senator T. L. Clingman, of Asheville, N. C. He wrote a paper on the same subject some months ago in Health and Home, of Washington, D. C., in which also we find the present paper. We give it a place in our journal, hoping that, with due allowance for the enthusiasm of the writer, it contains facts of practical value, and that our readers may learn the methods of using the article, and test, in their practice, the wonderful properties attributed to the Tobacco plant. We will be pleased to have reports of any experiments made in the use of the article in practice:—EDITOR RECORD.]

I am asked what kind of tobacco ought to be used, and how it is to be applied, and for what cases of injury or disease. With a view of meeting such inquiries and affording the information necessary to enable all persons properly to use this great remedy I will try to present a condensed statement.

I formerly used the ordinary manufactured tobacco in such cases as it could be had at the cigar stands in the hotels. A cake of this thrown into a bowl of cold water in an hour or less would become soft and spongy, and could be separated so that the leaves could be obtained, but, for reasons heretofore published in my original article, I do not advise that form of use. About three years ago I

ascertained by some experiments that, of late, tobacco manufacturers mix with their tobacco, for the purpose of improving its flavor, certain poisonous drugs, such as Tonka bean, wintergreen, and some other things. Though such tobacco may serve a beneficial purpose, yet I would prefer to use the leaf before it has been manufactured. Again, the darker leaves being stronger are better than the light yellow leaves. Also leaves of the tobacco cut last year are better than those freshly cut in this season, as tobacco seems to gather strength with age.

A bunch of these leaves thrown into a bowl of cold water will become moist and soft, so that the large stem in the center may be taken out. Hot water will answer the purpose sooner than cold, but either will do.

When this has been done, not less than two thicknesses of the leaf should be placed directly on the part to be relieved. As, however, the heat of the skin tends to dry the tobacco in a few minutes a wet bandage must be laid over it. About four thicknesses of common white cotton cloth will be sufficient, but this should be well soaked in the water before it is put on, then a bandage of the same cloth may be laid over it, and water, from time to time, should be applied by pressing a wet rag on it so as to keep the tobacco moist.

Tobacco, in this manner, may be applied directly on a cut or a wound, or any raw place without injury. The first effect of the tobacco, however, is stimulating or slightly irritating, but this after a few minutes ceases and the sedative effect soon comes on. When, for example, the tobacco is placed on the eye, as a little of the juice gets in between the eyelids some smarting or itching is felt, but after a few minutes this ceases, and no more effect is produced on the feelings than if a wet cloth were kept over the eye.

Within less than an hour the sedative effect is perceived, and within two hours, usually, all external inflammations are reduced. Obstinate cases, of course, require longer applications.

Most persons sleep under the influence of tobacco, but some do not, just as morphine causes some persons to sleep while others are wakeful. When I have tobacco on it is impossible for me to sleep, but, instead, unusual mental activity is produced, which continues throughout the next day following a night's application.

As long as there is inflammation about the part no nausea will be felt. As soon, however, as nausea is perceived the poultice may be removed, as the sedative effect has been produced. After the removal of the bandage the nausea passes away in a short time.

When one wishes to cure a bunion or a corn, after the tobacco has been applied as above directed, it is easy to get the sock over it, and by moistening the sock from time to time a cure is usually effected in a single night.

I will now proceed to refer to such cases as have been cured by the use of tobacco. In making this statement I shall only mention cases that I have observed myself, or such as have been stated by persons of good character and standing. If I were to give their statements at length a volume would be required to contain them. I therefore only give undoubted facts.

To begin with the head, I may say that all cases of erysipelas, whether on the head or face, or any other part of the body, are cured. In some cases where the head was swollen to almost double its usual size, and the patient was supposed by the attending physician about to die, an application of tobacco effected a complete cure.

Again, all cases of sore eyes, whether caused by injury or of disease, and whether old cases or fresh ones, have been cured. In some cases where there was total blindness a cure was at once effected, and the sight restored perfectly.

In the third place, all wounds, whether cuts, bruises, or contusions, have been easily cured. Sprains of the knee or ankle joints, where they were swelled to double the natural size, have been completely cured by a single night's application. Old cases, where the patient had suffered for months and years, have been cured.

In all cases where the remedy was tried, bunions and corns on the feet have been cured, even where, in some cases, they were said to be forty years old.

All cases of sore throat are cured, whether caused by diphtheria, croup, scarlet fever or quinsy. In more than one instance the patient was cured when seemingly at the point of death, and the case pronounced hopeless by the attending physicians.

All bone felons have been cured usually by a single night's application of the tobacco.

I have been informed of a number of cases in which the tobacco was applied as a remedy for hemorrhoids, and in every instance a single night's application is represented to have effected a cure. If tobacco should be applied to a wound neither mortification nor lock-jaw would ever supervene. In one case of lockjaw where the surgeon had pronounced the case hopeless, according to the published statement of a gentleman, a cure was effected by the application of a tobacco poultice to the stomach.

For cholera morbus, an application of tobacco to the stomach gives relief. A Senator told me that, when suffering from constipation most terribly, he had two physicians with him for four days and nights, with no advantage from their remedies, and when the pain became so intolerable that he felt that he would not get through the night, remembering what I had told him about tobacco, he caused it to be applied to his side and back, and in half an hour he was relieved, and immediately recovered.

Again, a great many cases of neuralgia, whether the case was accompanied with inflammation or not, have been cured by tobacco. In one case the patient said his eyes was so much inflamed that it seemed about to burst, and the application effected a complete cure.

Physicians here tell me all cases of orchitis are cured by tobacco, and usually in one night.

All kinds of sores on the feet can be immediately cured. Mr. Justice, the county surveyor here, after suffering for eight years with a very sore foot, was completely cured by a single night's application. In like manner Mr. Blair, who had lain in bed for many weeks with a foot so painful and swollen that he could bear no cover on it, was immediately relieved. Many cases similar to these have been mentioned to me.

I will now refer to other classes of cases which are supposed to be internal or general to the system. Col. J. H. Rumbough, the proprietor of the large establishment at the Warm Springs, about eight years ago, had, from overheat, a partial sunstroke. From that day, for three or four years, he every day suffered with a dull headache, which at times was very painful. Last week, when at this place, he told me that four years ago, when he met me in Washington City, I advised him to put a tobacco poultice on the back of his head, explaining particularly how it should be used ; that he tried it on the night following, and on the next day found that the pain was gone, and that, though four years have been passed, there has been no return of the pain.

Many cases of rheumatism have also been cured in this vicinity, some very acute cases, while others were of long standing. Also cases of gout, and rheumatic gout, have been invariably relieved, and with no evil effect on other parts of the system. It has been often stated that when gout was relieved by wet cloths, or some other soothing applications, it has been transferred to some other part of the system, and in some instances attacked the stomach or heart with fatal results. But when it has been relieved by a tobacco application, in no instance that I have heard of has there been any subsequent evil result. This I attribute to the penetrative power of the tobacco. While it drives away the inflammation at the place of its application, it is a most powerful nerve stimulant, and its influence is extended over the whole system. When applied to the head, or any part of the body, within two hours its effect will be perceived by a relaxing influence on the lower intestines and urinary organs. Morphine, in small doses, with many persons, stimulates the nerves, but causes constipation, while tobacco, though it is even a greater stimulant to the nerves, is an aperient to the intestinal canal and urinary organs. I have found that when I had a large plaster of tobacco on my hip and loin to relieve sciatica, a sensation was excited in old wounds and sores about the feet and legs, as well as the influence felt on the intestines. It ought to be stated that in two attacks of sciatica which I have experienced, tobacco cured me after the remedies of the doctors failed to help me.

I formerly stated to you that Mr. J. S. Carter, of this county, who had for two years suffered from dropsy, from head to foot, and who was regarded by his physicians as beyond recovery, at the suggestion of his mother, who had read my first article on the tobacco cure, applied it to his legs, and in a few nights was completely cured, and has had no return. Though three months have since passed he has had no return of the dropsy.

A stout gentleman in this county told me that he had a violent attack of pleurisy, which gave him a severe pain in his side, which was also considerably swollen. He decided to apply tobacco to his side, and was so entirely cured that on the second day thereafter he came into town and stated his experience to me.

I now give you a published statement of Dr. J. S. T. Baird, a physician of high standing and great experience in this vicinity :

From the Asheville (N. C.) Citizen.

MORE OF THE TOBACCO REMEDY.

Hon. T. L. CLINGMAN :

DEAR SIR—I cheerfully comply with your request and give you the results of my experience and observations with regard to the claims of your recent great discovery, and of its virtues as a remedial agent. It gives me great pleasure to add my testimony, most unhesitatingly, to that of the many others who are giving it such unqualified praise.

I have recently had occasion to test its efficacy in a number of cases—some of which I will give you—and in every one of which it gave entire satisfaction, and accomplished the ends for which it was used. The first was my own, which was a protracted spell of most distressing nervous headache, which lasted some days, and would not yield to the usual remedies. An application to the forehead and temples of some large tobacco leaves folded together and well moistened with warm water gave prompt and perfect relief. Another case was that of a most worrying and harrassing cough of some duration, brought on by wet feet and exposure to dampness and night air. Some of the most approved syrups failed to give relief, but a few tobacco leaves saturated with warm water and applied to the chest over the region of the lungs and about the throat gave speedy and permanent relief. Another and most interesting case was that of my own little four-year old daughter, who was most violently attacked with that dreadful and fell destroyer diphtheria, the disease developing with such alarming rapidity as to awaken in me the deepest solicitude. I made haste to apply the tobacco, and had not long to wait until the beneficial effects were perfectly apparent. A few tobacco leaves prepared as in the cases above stated and applied to the throat very soon checked and modified the symptoms as to render the disease easily tractable, and her convalescence was rapid and steady. Of course, I used other remedies, but they were only snch as had failed in many less marked cases than hers. I believe that her recovery is mainly attributable to the tobacco. Still another case is that of my neighbor, Mr. N. B. Westall, who had a violent attack of diphtheria. * * * *

Mr. Bristol, the gentleman who has charge of the office in this hotel (the Eagle), had, for at least a dozen years, at intervals, been a sufferer from attacks of pain in the side, and indigestion, attributed to torpidity of the liver. Recently one of those attacks came on ; he applied tobacco to the seat of the pain—the region of the liver—and a single night's application took away all the pain. He has had no return of the pain since and his digestion is good. He was also suffering, lately, much pain from a contraction of the sinews and muscles under his knee, but an application of tobacco for one night removed the pain. He could straighten his leg and walk as usual, seeming entirely well. He vows that no consideration would induce him to give up tobacco.

A gentleman who brought his wife to this region for cure of consumption, says that, as she was lately suffering from pain in the side, he applied tobacco and next day the pain was relieved and she felt better.

A lady, not far from this place, whose husband has been a member of our legislature, suffered from what is called milk leg. Her hip, thigh, and leg were much swollen. After weeks of suffering, the physicians pronounced it a case of pyæmia or blood-poisoning. Her husband caused tobacco to be applied, and he stated to two gentlemen, who informed me, that he regarded her as well. Cases of what is properly known as fever cake, of long standing, have been removed at once by tobacco.

Persons have told me that cases of cancer have also been cured, but the sores may possibly not have been really cancers. I found, however, a case which the two physicians who practiced longest in this town, and are gentlemen of the highest standing, have pronounced cancer. The patient, Mr. John Ledford, had borne it seven years on the side of his nose. I saw him and observed a white scab and swelling, with redness around it, which extended to the eye and cheek. I advised him to try tobacco on it. I saw him after a week's trial. The inflammation around the scab had disappeared and the scab seemed smaller. When I last saw him the scab was smaller and seemed about to fall off and the sore to disappear. Sores inside the nose, attributed to catarrhal irritation, and of long standing, have been entirely cured by putting into the nostrils a plug of wet tobacco and keeping it there for a few hours at a time. I close with reference to cattle cases. In every instance in this region where tobacco in milk has been given to hogs suffering from cholera a cure has been the result. Even where the hog was unable to stand, a drenching with tobacco juice cured him. Milk sickness among the cattle is cured by tobacco.

Mr. Preston F. Patton, a large farmer in this county, informs me of other remarkable cures. In the spring of the year many of his cows died from some poisonous plant which came up on his plantation. He says that during last May at least twenty-five of these fell down and would have died, but that he drenched them with about a pint of tobacco juice, and in less than half an hour every one of them arose and recovered.

